# Supplementary figures and images for: Frequency switching between oscillatory homeostats and the regulation of p53
Source: PLoS One. 2020 May 20;15(5):e0227786. doi: 10.1371/journal.pone.0227786 (PMC7239446; doi:10.1371/journal.pone.0227786)

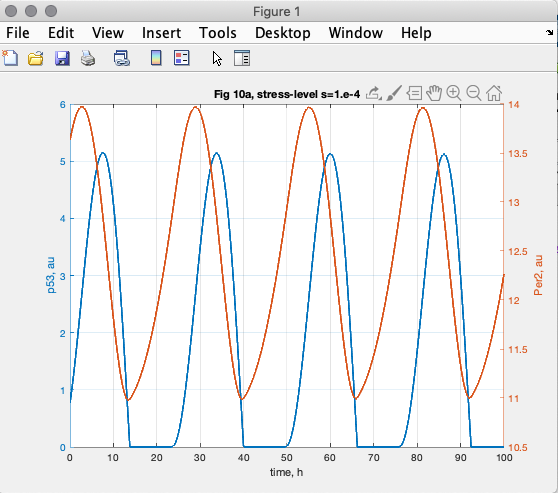

Supplement: S1 Matlab — A zip-file with Matlab programs showing the results from Figs 5a, 5b, 10a–10d and 11a–11c (left panels). (ZIP) [file pone.0227786.s001.zip › fig10a_matlab/Screenshot 2020-03-23 at 10.51.37.png]

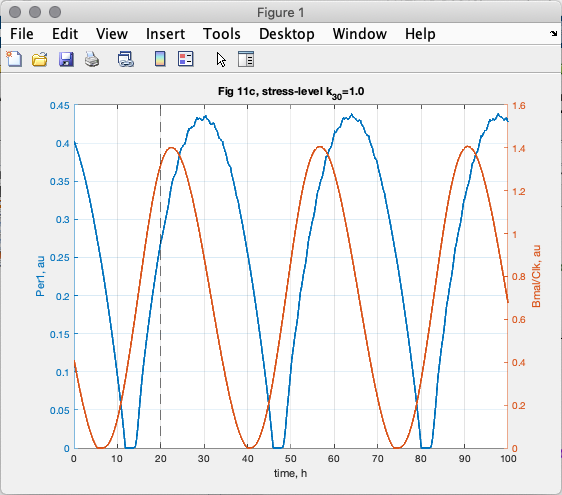

Supplement: S1 Matlab — A zip-file with Matlab programs showing the results from Figs 5a, 5b, 10a–10d and 11a–11c (left panels). (ZIP) [file pone.0227786.s001.zip › fig11c_matlab/Screenshot 2020-03-23 at 11.10.28.png]

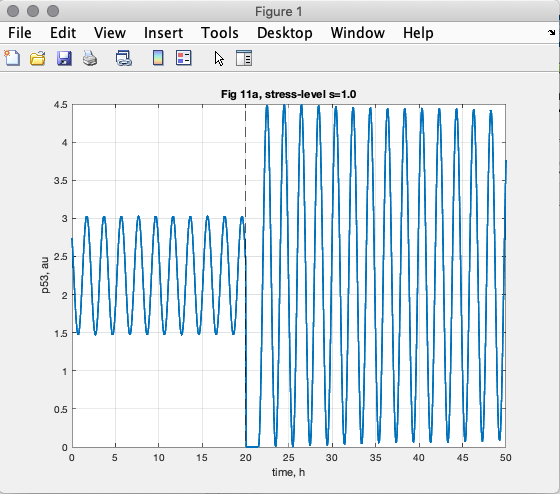

Supplement: S1 Matlab — A zip-file with Matlab programs showing the results from Figs 5a, 5b, 10a–10d and 11a–11c (left panels). (ZIP) [file pone.0227786.s001.zip › fig11a_matlab/Screenshot 2020-03-23 at 11.06.13.png]

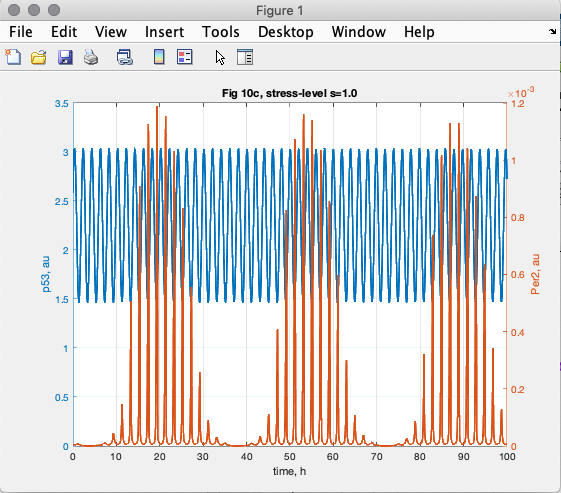

Supplement: S1 Matlab — A zip-file with Matlab programs showing the results from Figs 5a, 5b, 10a–10d and 11a–11c (left panels). (ZIP) [file pone.0227786.s001.zip › fig10c_matlab/Screenshot 2020-03-23 at 10.56.37.png]

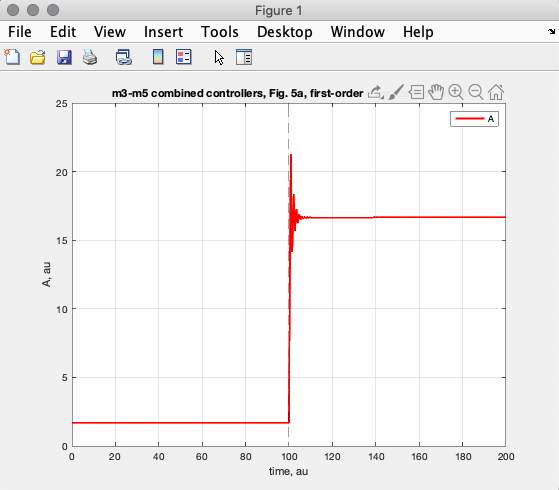

Supplement: S1 Matlab — A zip-file with Matlab programs showing the results from Figs 5a, 5b, 10a–10d and 11a–11c (left panels). (ZIP) [file pone.0227786.s001.zip › fig5a_matlab/Screenshot 2020-03-23 at 10.44.07.png]

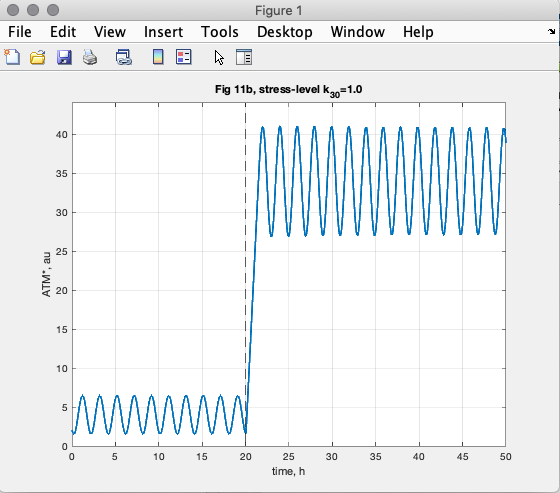

Supplement: S1 Matlab — A zip-file with Matlab programs showing the results from Figs 5a, 5b, 10a–10d and 11a–11c (left panels). (ZIP) [file pone.0227786.s001.zip › fig11b_matlab/Screenshot 2020-03-23 at 11.07.55.png]

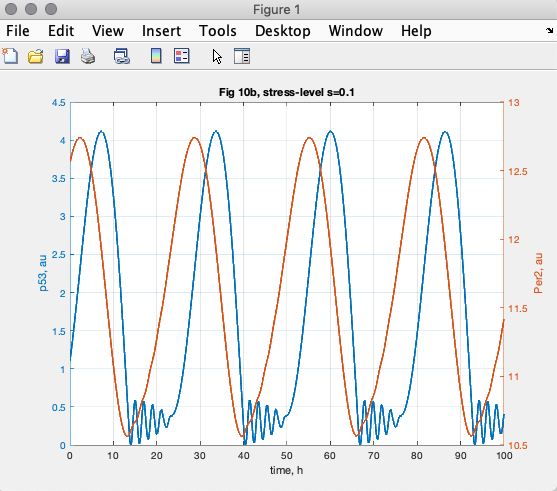

Supplement: S1 Matlab — A zip-file with Matlab programs showing the results from Figs 5a, 5b, 10a–10d and 11a–11c (left panels). (ZIP) [file pone.0227786.s001.zip › fig10b_matlab/Screenshot 2020-03-23 at 10.54.00.png]

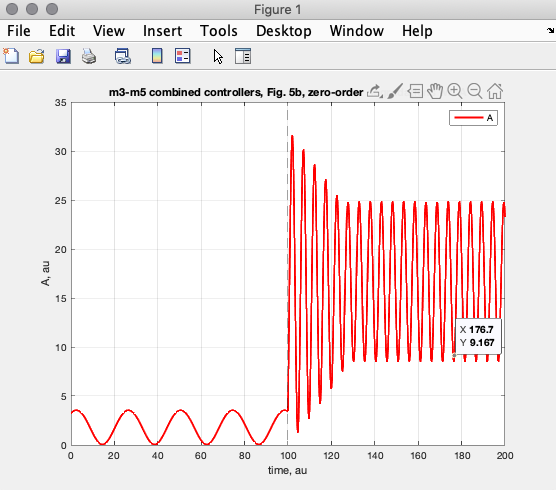

Supplement: S1 Matlab — A zip-file with Matlab programs showing the results from Figs 5a, 5b, 10a–10d and 11a–11c (left panels). (ZIP) [file pone.0227786.s001.zip › fig5b_matlab/Screenshot 2020-03-23 at 10.48.28.png]

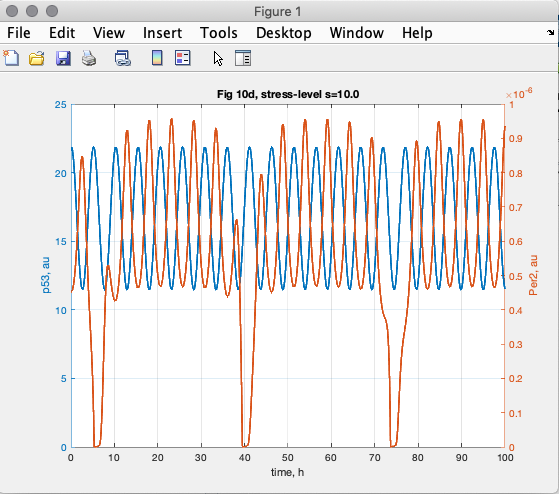

Supplement: S1 Matlab — A zip-file with Matlab programs showing the results from Figs 5a, 5b, 10a–10d and 11a–11c (left panels). (ZIP) [file pone.0227786.s001.zip › fig10d_matlab/Screenshot 2020-03-23 at 11.02.02.png]
