# Supplementary material for: Frequency switching between oscillatory homeostats and the regulation of p53
Source: PLoS One. 2020 May 20;15(5):e0227786. doi: 10.1371/journal.pone.0227786 (PMC7239446; doi:10.1371/journal.pone.0227786)
Supplement: S1 Text — (PDF) [file pone.0227786.s004.pdf]

Supporting Material, File S1 Text

Frequency switching between oscillatory homeostats and the regulation of p53

P. Ruoff[1*], N. Nishiyama[2]
[1]Department of Chemistry, Bioscience, and Environmental Engineering
[2]Division of Mathematical and Physical Sciences
Graduate School of Natural Science and Technology
Kanazawa University, Kanazawa, Japan

*Corresponding author. Address: Department of Chemistry, Bioscience, and Environmental Engineering, University of Stavanger, Stavanger, Norway, Tel.: (47) 5183-1887, E-mail: peter.ruoff@uis.no

Harmonic approximation of period lengths and amplitudes of oscillatory m3 and m5 controllers

Determination of period P_{m5} in oscillatory m5 controller

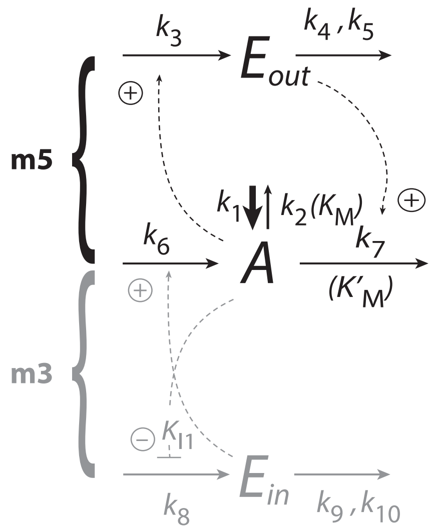

Figure S1. Controller motif m5 with A and E_{out} as the controlled and controller variables, respectively. The grayed m3 controller is not considered, i.e., E_{in} is neglected. However, we still allow a constitutive (constant) inflow to A via rate constant k_6. Since m5 is an outflow controller m5 compensates for inflow perturbations which are indicated by thick arrow k_1.

The rate equations for A and E_{out} are:

$$\dot{A} = k_1 + k_6 - \frac{k_2 \cdot A}{K_M + A} - \frac{k_7 \cdot A \cdot E_{out}}{K'_M + A} \tag{S1}$$

$$\dot{E_{out}} = k_3 \cdot A - \frac{k_4 \cdot E_{out}}{k_5 + E_{out}} \tag{S2}$$

Integral control is introduced by the condition $k_5 \ll E_{out}$, such that

$$\frac{E_{out}}{k_5 + E_{out}} \cong 1 \tag{S3}$$

Because $<\dot{E_{out}}>=0$ during steady state oscillations, the oscillator's set-point, A_{set}^{m5}, is determined by:

$$<\dot{E_{out}}>=k_3<A>-k_4\left\langle\frac{E_{out}}{k_5+E_{out}}\right\rangle\cong k_3<A>-k_4=0 \quad\Rightarrow\quad <A>\cong A_{set}^{m5}=\frac{k_4}{k_3} \tag{S4}$$

Oscillations in controller m5 occur, because of the conditions $K_M\ll A$ and $K_M^{'}\ll A$, which lead to zero-order degradation rates with respect to A.

To derive the period length, Eq S1 is approximated by

$$\dot{A} = k_1 + k_6 - k_2 - k_7{\cdot}E_{out} \tag{S5}$$

As a caveat, even when using zero-order conditions in the degradation of A, Eq S5 should not be used instead of Eq S1 in numerical calculations, because from Eq S5 negative A values may result.

Taking the time-derivative of Eq S5 and using the approximation of Eq S3 leads to

$$\ddot{A} = -k_7{\cdot}\dot{E_{out}} = -k_7(k_3{\cdot}A - k_4) \tag{S6}$$

Rearranging Eq S6 gives the equation of a harmonic oscillator

$$\frac{\ddot{A}}{k_3k_7} + A = \frac{\ddot{A}}{\omega^2} + A = \frac{k_4}{k_3} = A_{set}^{m5} \tag{S7}$$

where A oscillates sinusoidally around the set-point A_{set}^{m5} with frequency ω

$$A(t) = A_{ampl}^{m5}{\cdot}\sin(\omega{\cdot}t+\phi) + A_{set}^{m5} \tag{S8}$$

and period length P_{m5}

$$P_{m5} = \frac{2\pi}{\omega} = \frac{2\pi}{\sqrt{k_3k_7}} \tag{S9}$$

Determination of A and $\mathbf{E_{out}}$ amplitudes for oscillatory m5 controller

A-amplitude $\mathbf{A^{m5}_{ampl}}$

The m5 oscillator is *conservative*, which means that an energy function H (Hamiltonian) can be found, which is able to describe the trajectories of the oscillator in analogy to the Hamilton-Jacobi equations (see Ref (1) and the Supporting Information of Ref (2)).

In case of the m5 oscillator the H-function is given as

$$H_5(A, E_{out}) = -\int (\dot{A})\, dE_{out} + \int (\dot{E_{out}})\, dA \tag{S10}$$

Inserting the expressions of $\dot{A}$ (Eq S5) and $\dot{E_{out}}$ (with the approximation of Eq S3) we get

$$H_5(A, E_{out}) = -\int (k_1 + k_6 - k_2 - k_7{\cdot}E_{out})\, dE_{out} + \int (k_3{\cdot}A - k_4)\, dA \tag{S11}$$

Integrating Eq S11 leads to

$$\begin{aligned} H_5(A, E_{out}) &= a{\cdot}(E_{out})^2 - b{\cdot}E_{out} + c{\cdot}A^2 - d{\cdot}A \\ &= a\left(E_{out} - \frac{b}{2a}\right)^2 + c\left(A - \frac{d}{2c}\right)^2 - \alpha \end{aligned} \tag{S12}$$

where $a{=}½{\cdot}k_7$, $b{=}k_1{+}k_6{-}k_2$, $c{=}½{\cdot}k_3$, $d{=}k_4$, and $\alpha{=}\frac{d^2}{4c}{+}\frac{b^2}{4a}$.

The equation

$$H_5(A, E_{out}) = H_5(A_0, E_{out,0}) = H_{5,0} \tag{S13}$$

defines an ellipse as the trajectory of the oscillations in $A{-}E_{out}$ space going through the point $(A_0, E_{out,0})$ with $d/2c$ as the set-point of A (A^{m5}_{set}). Fig S2 shows the geometrical arrangement.

The amplitudes of A and E_{out} for a given trajectory defined by point $(A_0, E_{out,0})$ can be calculated by the difference between the maximum and minimum A and E_{out} values (Fig S2). A_{max} and A_{min} values are obtained when setting in Eq S13 E_{out}=$b/2a$, which leads to

$$A_{max} = \frac{d}{2c} + \sqrt{\frac{H_{5,0}+\alpha}{c}} \tag{S14}$$

and

$$A_{min} = \frac{d}{2c} - \sqrt{\frac{H_{5,0}+\alpha}{c}} \tag{S15}$$

with the amplitude of A, A_{ampl}, given by

$$A^{m5}_{ampl} = A_{max} - A_{min} = 2 \times \sqrt{\frac{H_{5,0}+\alpha}{c}} \tag{S16}$$

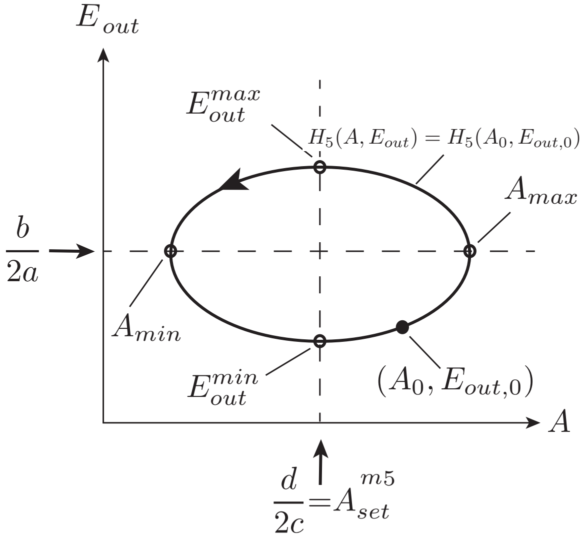

Figure S2. Eq S13 describes an ellipse as trajectories for the oscillatory m5 controller. The initial concentration $(A_0, E_{out,0})$ defines the ellipse.

$\mathbf{E_{out}}$-amplitude $\mathbf{E_{out}^{ampl}}$

Likewise, the amplitude of E_{out} is calculated by setting in Eq S13 $A{=}d/2c$ which gives us, by the resulting quadratic equation, the values

$$E_{out}^{max} = \frac{b}{2a} + \sqrt{\frac{H_{5,0}{+}\alpha}{a}} \tag{S17}$$

and

$$E_{out}^{min} = \frac{b}{2a} - \sqrt{\frac{H_{5,0}{+}\alpha}{a}} \tag{S18}$$

with the amplitude of E_{out}, E_{out}^{ampl}, given by

$$E_{out}^{ampl} = E_{out}^{max} - E_{out}^{min} = 2 \times \sqrt{\frac{H_{5,0}{+}\alpha}{a}} \tag{S19}$$

Fig S3 shows a comparison between numerical and harmonic approximation of the oscillatory controller m5 with rate parameters from Fig 5d. In addition, Table 1 compares the numerically and analytically calculated period lengths and A, E_{out} amplitudes as k_1 increases.

TABLE 1: Comparison between numerical results and the analytically calculated period lengths and amplitudes for oscillatory controller m5 as a function of k_1. Other parameter values as in Fig S3.

k_1	P_{num}	P_{anal}	A_{ampl}^{num}	A_{ampl}^{anal}	$E_{out}^{ampl,num}$	$E_{out}^{ampl,anal}$
5.0	5.13	5.13	8.16	7.03	20.00	17.22
10.0	5.13	5.13	15.85	16.14	38.80	39.50
20.0	5.13	5.13	21.69	22.82	53.12	55.90
40.0	5.13	5.13	30.51	30.72	74.73	75.26

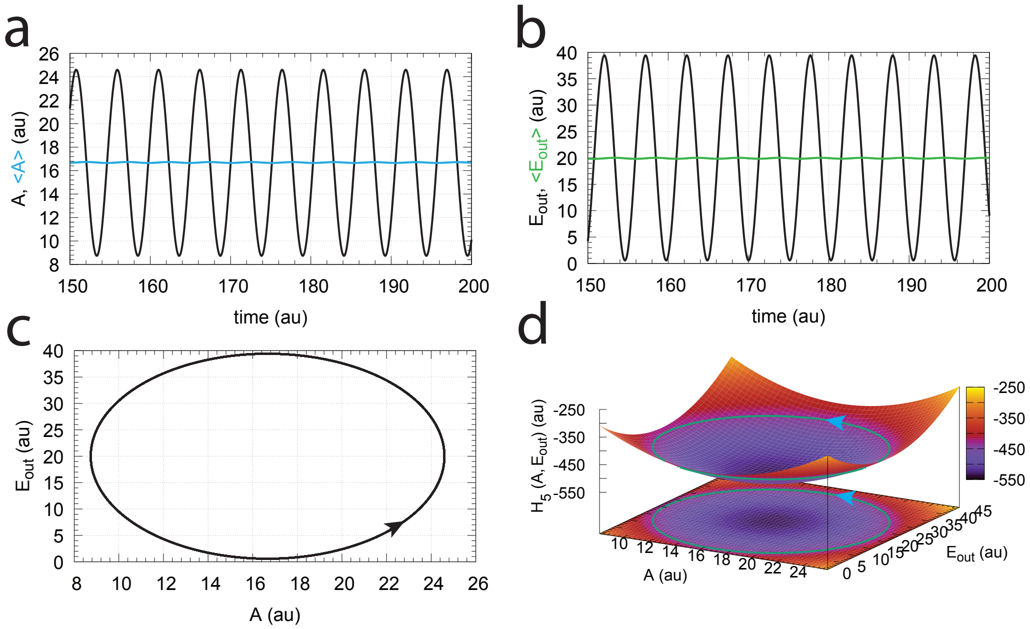

Figure S3. Comparison between numerical and harmonic approximation of oscillatory controller m5. Panels a and b show the numerically calculated oscillations in A, $<A>$, E_{out}, and $<E_{out}>$ by solving Eqs 1-3 in main article. Panel c shows the corresponding trajectory in the $E_{out}-A$ phase space. Panel d shows the $H_5(A, E_{out})$ surface (and its projection into the $E_{out}-A$ phase space) as a function of A and E_{out}. The trajectory on the surface is defined by Eq S13 through the initial concentrations $(A_0, E_{out,0})$ via $H_{5,0}$ and is an approximation to the numerically calculated trajectory in panel c. Rate constants: k_1=10.0, k_2=0.0, k_3=3.0, k_4=50.0, $k_5=1 \times 10^{-8}$, k_6=0.7, k_7=0.5, k_8=1.2, k_9=1.0, $k_{10}=1 \times 10^{-6}$, $K_M, K_M^{'}$ both 1×10^{-6}, and K_{I1}=8.5. Initial concentrations: A_0=10.57, $E_{out,0}$=7.59, $E_{in,0}=1.15 \times 10^{-6}$.

Determination of period P_{m3} in oscillatory m3 controller

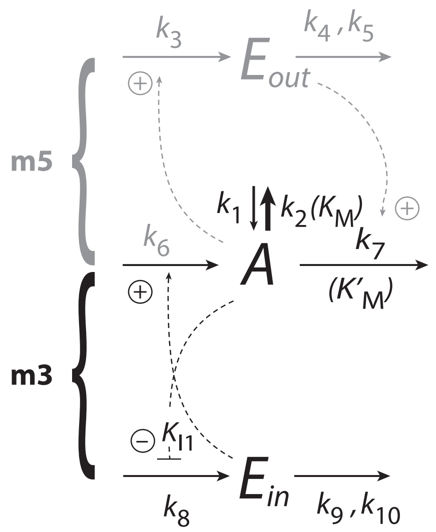

Figure S4. Controller motif m3 with A and E_{in} as the controlled and controller variables, respectively. The grayed m5 controller is not considered, i.e., E_{out} is neglected. However, we still allow a constitutive (constant) zero-order Michaelis-Menten outflow from A using rate constants k_7 and K_M'. As an inflow controller m3 compensates for outflow perturbations which are indicated by thick arrow k_2.

The rate equations for A and E_{in} are:

$$\dot{A} = k_1 - \frac{k_2 \cdot A}{K_M + A} + k_6 \cdot E_{in} - \frac{k_7 \cdot A}{K_M' + A} \tag{S20}$$

$$\dot{E_{in}} = \frac{k_8 \cdot K_{I1}}{K_{I1} + A} - \frac{k_9 \cdot E_{in}}{k_{10} + E_{in}} \tag{S21}$$

Using $\ddot{\mathbf{A}}$ in derivation of the period length $\mathbf{P_{m3}}$

Assuming zero-order conditions of the Michaelis-Menten expressions in Eq S20 such that

$$\frac{A}{K_M+A}=\frac{A}{K_M^{'}+A}\cong 1 \tag{S22}$$

Eq S20 is then written as:

$$\dot{A}=k_1-k_2+k_6{\cdot}E_{in}-k_7 \tag{S23}$$

Taking the second time-derivative of A and using Eq S21 gives

$$\ddot{A}=k_6\dot{E_{in}}=\frac{k_6k_8K_{I1}}{K_{I1}+A}-k_6k_9 \tag{S24}$$

Dividing Eq S24 by $\frac{k_6k_8K_{I1}}{K_{I1}+A}$ leads to

$$\begin{aligned}\frac{\ddot{A}}{\left(\frac{k_6k_8K_{I1}}{K_{I1}+A}\right)}&=1-\frac{k_6k_9}{\left(\frac{k_6k_8K_{I1}}{K_{I1}+A}\right)}=1-\frac{k_6k_9(K_{I1}+A)}{k_6k_8K_{I1}}\\&=1-\frac{k_6k_9K_{I1}}{k_6k_8K_{I1}}-\frac{k_6k_9A}{k_6k_8K_{I1}}\end{aligned} \tag{S25}$$

Rearranging Eq S25 leads to

$$\frac{\ddot{A}}{\left(\frac{k_6k_8K_{I1}}{K_{I1}+A}\right)}+\frac{k_6k_9A}{k_6k_8K_{I1}}=\frac{k_6k_8K_{I1}-k_6k_9K_{I1}}{k_6k_8K_{I1}} \tag{S26}$$

Multiplying Eq S26 by $(k_6k_8K_{I1})$, we get

$$\frac{\ddot{A}}{\left(\frac{1}{K_{I1}+A}\right)}+k_6k_9A=k_6K_{I1}(k_8-k_9) \tag{S27}$$

and finally, the harmonic-oscillator-type of equation

$$\frac{\ddot{A}}{\left(\frac{k_6k_9}{K_{I1}+A}\right)}+A=\frac{K_{I1}(k_8-k_9)}{k_9}=A_{set}^{m3} \tag{S28}$$

which is compared with

$$\frac{\ddot{A}}{\omega^2} + A = \frac{K_{I1}(k_8 - k_9)}{k_9} = A_{set}^{m3} \tag{S29}$$

By setting

$$\omega = \sqrt{\left(\frac{k_6 k_9}{K_{I1} + <A>}\right)} \tag{S30}$$

we get an approximative expression for the m3-oscillators frequency, where $<A>$ is the average value of A defined as

$$<A> = \frac{1}{\tau}\int_0^\tau A(t)\,dt \tag{S31}$$

The period length is calculated according to $P{=}2\pi/\omega$, i.e.,

$$P_{m3} = \frac{2\pi}{\sqrt{\left(\frac{k_6 k_9}{K_{I1} + <A>}\right)}} \tag{S32}$$

Using $\ddot{\mathbf{E}}_{\mathbf{in}}$ in derivation of the period length $\mathbf{P_{m3}}$

In this case we write Eq S21 as

$$\dot{E}_{in} = \frac{k_8 \cdot K_{I1}}{K_{I1} + A} - k_9 \tag{S33}$$

by using approximately the equality

$$\frac{E_{in}}{k_{10} + E_{in}} \cong 1 \tag{S34}$$

Calculating $\ddot{E}_{in}$ and inserting $\dot{A}$ (Eq S23) into the resulting equation gives:

$$\ddot{E}_{in} = -\frac{k_8 K_{I1}}{(K_{I1} + A)^2}\dot{A} = -\frac{k_8 K_{I1}}{(K_{I1} + A)^2}\,(k_1 - k_2 - k_7 + k_6 E_{in}) \tag{S35}$$

Rearranging Eq S35 we get

$$\ddot{E_{in}} + \frac{k_6 k_8 K_{I1} E_{in}}{(K_{I1}+A)^2} = \frac{k_8 K_{I1}(k_2 + k_7 - k_1)}{(K_{I1}+A)^2} \tag{S36}$$

and

$$\frac{\ddot{E_{in}}}{\frac{k_6 k_8 K_{I1}}{(K_{I1}+A)^2}} + E_{in} = \frac{k_2+k_7-k_1}{k_6} = <E_{in}> \tag{S37}$$

where $<E_{in}>$ is the average value of E_{in} defined as

$$<E_{in}> = \frac{1}{\tau}\int_0^\tau E_{in}(t)\, dt \tag{S38}$$

$<E_{in}>$ is determined by setting $<\dot{A}> = 0$ in Eq S23 and solving for $<E_{in}>$. By comparing Eq S37 with that of a harmonic oscillator

$$\frac{\ddot{E_{in}}}{\omega^2} + E_{in} = <E_{in}> \tag{S39}$$

oscillating around $<E_{in}>$, we can approximate the period to

$$P_{m3} = \frac{2\pi}{\omega} = \frac{2\pi}{\sqrt{\frac{k_6 k_8 K_{I1}}{(K_{I1}+<A>)^2}}} \tag{S40}$$

where $<A>$ is defined by Eq S31.

Setting Eq S40 equal to Eq S32 we see that the two equations are coupled by the relationship

$$<\dot{E_{in}}> = \frac{k_8 \cdot K_{I1}}{K_{I1}+<A>} - k_9 = 0 \tag{S41}$$

which describes the set-point for A of the oscillatory m3 controller in analogy to Eq. 5., i.e.,

$$A_{set}^{m3} = <A> = \frac{K_{I1}(k_8-k_9)}{k_9} \tag{S42}$$

Determination of $\mathbf{E_{in}}$ and A amplitudes for oscillatory m3 controller

$\mathbf{E_{in}}$-amplitude $\mathbf{E_{in}^{ampl}}$

The rate equations S20 and S21 are rewritten as

$$\dot{A} = k_1 - k_2 + k_6{\cdot}E_{in} - k_7 \tag{S43}$$

$$\dot{E_{in}} = \frac{k_8{\cdot}K_{I1}}{K_{I1}{+}A} - k_9 \tag{S44}$$

by using

$$\frac{A}{K_M{+}A} = \frac{A}{K_M'{+}A} \cong 1 \tag{S45}$$

and

$$\frac{E_{in}}{k_{10}{+}E_{in}} \cong 1 \tag{S46}$$

The m3 Hamitonian $H_3(A, E_{in})$ takes the form

$$\begin{aligned} H_3(A, E_{in}) &= -\int \dot{A}\, dE_{in} + \int \dot{E_{in}}\, dA \\ &= -\int \left[k_6{\cdot}E_{in}{-}(k_2{+}k_7{-}k_1)\right] dE_{in}{+}\int \left[\left(\frac{k_8K_{I1}}{K_{I1}{+}A}\right) {-}k_9\right] dA \\ &= -\frac{k_6E_{in}^2}{2}{+}(k_2{+}k_7{-}k_1)E_{in}{+}k_8K_{I1}\ln(K_{I1}{+}A){-}k_9A \end{aligned} \tag{S47}$$

The (conservative) m3 oscillations going through point $(A_0, E_{in,0})$ in the E_{in}-A phase space are described by the condition

$$H_3(A, E_{in}) = H_3(A_0, E_{in,0}) = H_{3,0} \tag{S48}$$

Fig S5 gives a schematic representation of the oscillation in the E_{in}-A phase space. When inserting $A{=}A_{set}^{m3}{=}K_{I1}(k_8{-}k_9)/k_9$ into Eq S48 a quadratic equation in E_{in} is obtained with the solutions

$$E_{in}^{min} = \frac{b}{2a} - \frac{\sqrt{b^2{-}4ac}}{2a} \tag{S49}$$

$$E_{in}^{max} = \frac{b}{2a} + \frac{\sqrt{b^2-4ac}}{2a} \tag{S50}$$

where a=0.5k_6, b=k_2+k_7−k_1, and c=$k_8 K_{I1} \ln(K_{I1}+A_{set}^{m3})-k_9 A_{set}^{m3}-H_{3,0}$

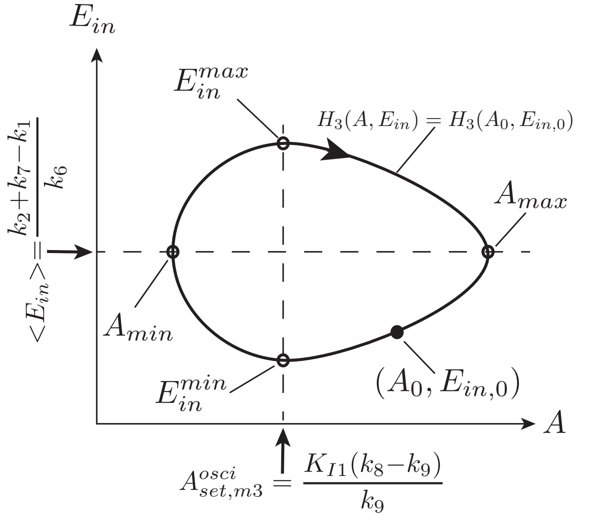

Figure S5. Schematic representation of a m3 trajectory in E_{in}-A phase space going through point $(A_0, E_{in,0})$.

The amplitude of the E_{in} oscillations, E_{in}^{ampl}, is then calculated as the difference between E_{in}^{max} and E_{in}^{min}

$$E_{in}^{ampl} = E_{in}^{max} - E_{in}^{min} = \frac{\sqrt{b^2-4ac}}{a} \tag{S51}$$

A-amplitude $\mathbf{A_{ampl}^{m3}}$

The amplitude of A for the oscillatory m3 controller is calculated via the determined A_{max} and A_{min} values when setting E=$<E_{in}>$ into Eq S48 (Fig S5), i.e.

$$H_3(A, <E_{in}>) = H_3(A_0, E_{in,0}) = H_{3,0} \tag{S52}$$

Eq S52 takes the form

$$c_1 + c_2 \ln(K_{I1}+A) - k_9 A = 0 \tag{S53}$$

where c_1=$-0.5k_6$<E_{in}>$^2 + (k_2$+k_7−k_1)<E_{in}> − $H_{3,0}$ and c_2=k_8K_{I1}. Eq S53 was solved numerically with Matlab thereby providing values for A_{max} and A_{min}. Fig S6 shows numeral calculations alongside with the corresponding H_3 surface and the oscillatory state when H_3=$H_{3,0}$ (Eq S48). Table 2 compiles the results of period lengths and amplitudes from numerical and analytical calculations. Numerical and analytical calculations show reasonable good agreements.

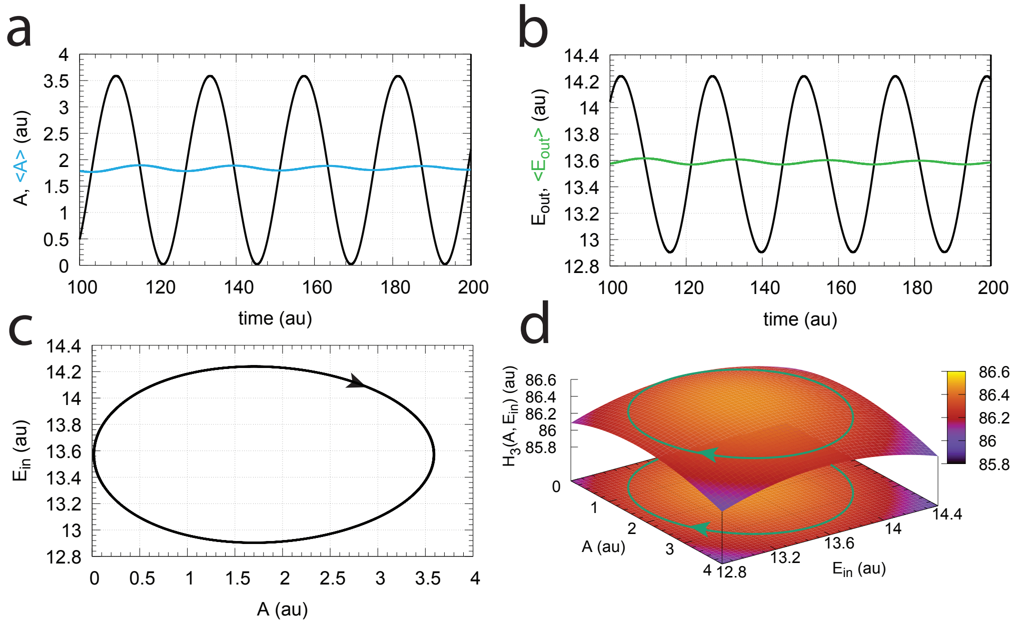

Figure S6. Comparison between numerical and harmonic approximation of oscillatory controller m3. Panels a and b show the numerically calculated oscillations in A, <A>, E_{in}, and <E_{in}> by solving Eqs 1-3 in main article. Panel c shows the corresponding trajectory in the E_{in}−A phase space. Panel d shows the $H_3(A, E_{in})$ surface (and its projection into the E_{in}−A phase space) as a function of A and E_{in}. The trajectory on the surface is defined by Eq S48 through the initial concentrations via $H_3(A_0, E_{in,0})$ and is an approximation to the numerically calculated trajectory in panel c. Rate constants: k_1=1.0, k_2=10.0, k_3=0.0, k_4=0.0, k_5=1×10^{-8}, k_6=0.7, k_7=0.5, k_8=1.2, k_9=1.0, k_{10}=1×10^{-6}, $K_M, K_M^{'}$ both 1×10^{-6}, and K_{I1}=8.5. Initial concentrations: A_0=3.22, $E_{out,0}$=1.0 (kept constant), $E_{in,0}$=13.22.

TABLE 2: Comparison between numerically and analytically calculated period lengths and amplitudes for oscillatory controller m3 as a function of k_2. Other parameter values as in Fig S6.

k_2	P_{num}	P_{anal}	A_{ampl}^{num}	A_{ampl}^{anal}	$E_{in}^{ampl,num}$	$E_{in}^{ampl,anal}$
5.0	24.01	24.20	3.60	3.60	1.35	1.35
10.0	24.01	24.19	3.49	3.46	1.34	1.33
20.0	24.01	24.28	3.60	3.60	1.35	1.35
40.0	24.01	24.24	3.57	3.55	1.33	1.33

References

[1] Drengstig, T.; Ni, X.; Thorsen, K.; Jolma, I.; Ruoff, P. *The Journal of Physical Chemistry B* **2012**, *116*(18), 5355–5363.

[2] Thorsen, K.; Agafonov, O.; Selstø, C. H.; Jolma, I. W.; Ni, X. Y.; Drengstig, T.; Ruoff, P. *PLOS ONE* **2014**, *9(9)*, e107766. doi:10.1371/journal.pone.0107766.
