# Supplementary material for: Frequency switching between oscillatory homeostats and the regulation of p53
Source: PLoS One. 2020 May 20;15(5):e0227786. doi: 10.1371/journal.pone.0227786 (PMC7239446; doi:10.1371/journal.pone.0227786)
Supplement: S2 Text — (PDF) [file pone.0227786.s005.pdf]

Supporting Material, File S2 Text

Frequency switching between oscillatory homeostats and the regulation of p53

P. Ruoff[1*], N. Nishiyama[2]
[1]Department of Chemistry, Bioscience, and Environmental Engineering
[2]Division of Mathematical and Physical Sciences
Graduate School of Natural Science and Technology
Kanazawa University, Kanazawa, Japan

*Corresponding author. Address: Department of Chemistry, Bioscience, and Environmental Engineering, University of Stavanger, Stavanger, Norway, Tel.: (47) 5183-1887, E-mail: peter.ruoff@uis.no

Determination of set-point and period length of the ATM* controller

Fig S1 shows the p53-ATM* negative feedback loop when ATM* is upregulated by DNA damage. With respect to p53 as the controlled variable we have a motif 1 negative feedback loop (1). The active (phosphorylated) form of ATM (ATM*) activates p53 via CHK2 (checkpoint kinase 2) (2, 3), while p53 dephosphorylates ATM* via the activation of the phosphatase WIP1 (3–5).

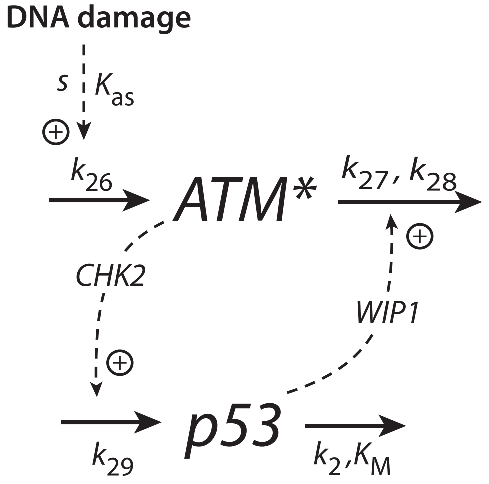

Figure S1. The feedback loop between p53 and ATM*. Symbol s represents the stress level and K_{as} is an activation constant.

When the stress level s is between 0.2 and 1.0 ATM* is the dominating regulator of p53 (Fig 9) and the rate equations for ATM* and p53 can be written as:

$$\dot{ATM^*} = \frac{k_{26} \cdot s}{K_{as}+s} - \left(\frac{k_{27} \cdot ATM^*}{k_{28}+ATM^*}\right) \cdot p53 \tag{S1}$$

$$\dot{p53} = k_{29} \cdot ATM^* - \frac{k_2 \cdot p53}{K_M+p53} \tag{S2}$$

By setting Eq S1 to zero, the set-point for p53 ($p53_{set}^{ATM^*}$) is calculated to

(see also Eq 24):

$$p53_{set}^{ATM^*} = \left(\frac{k_{26}}{k_{27}}\right) \cdot \left(\frac{s}{K_{as} + s}\right) \tag{S3}$$

The dependence/change of $p53_{set}^{ATM^*}$ as a function of the stress level s is an example of rheostatic regulation (6) where the set-point changes with the stress level and is defended towards increasing degradation rates k_2 (Fig 11).

When the ubiquitin-independent proteasomal degradation of p53 (via NQO1,(7–9)) is considered to be zero-order (low K_M, Fig S1) the system is described as a harmonic oscillator and the period can be calculated by the double time derivative of p53, $p\ddot{5}3$, i.e.,

$$p\ddot{5}3 = k_{29} A\dot{T}M^* = k_{29}\left(\frac{k_{26} \cdot s}{K_{as}+s}\right) - k_{27}k_{29}p53 \tag{S4}$$

Eq S4 can be written in form of the following equation:

$$\frac{p\ddot{5}3}{\omega^2} + p53 = \left(\frac{k_{26}}{k_{27}}\right) \cdot \left(\frac{s}{K_{as} + s}\right) = p53_{set}^{ATM^*} \tag{S5}$$

with $\omega^2 = k_{27}k_{29}$.

The solution of Eq S5 is

$$p53(t) = p53_{set}^{ATM^*} + A_{ampl}\sin(\omega \cdot t + \phi) \tag{S6}$$

Thus, $p53(t)$ oscillates with period

$$P_{p53}^{ATM^*} = \frac{2\pi}{\omega} = \frac{2\pi}{\sqrt{k_{27}k_{29}}} \tag{S7}$$

and amplitude A_{ampl} around its set-point $p53_{set}^{ATM^*}$.

References

[1] Drengstig, T.; Jolma, I.; Ni, X.; Thorsen, K.; Xu, X.; Ruoff, P. *Biophys J* **2012**, *103*(9), 2000–2010.

[2] Batchelor, E.; Mock, C. S.; Bhan, I.; Loewer, A.; Lahav, G. *Molecular Cell* **2008**, *30*(3), 277–289.

[3] Batchelor, E.; Loewer, A.; Lahav, G. *Nature Reviews Cancer* **2009**, *9*(5), 371.

[4] Fiscella, M.; Zhang, H.; Fan, S.; Sakaguchi, K.; Shen, S.; Mercer, W. E.; Woude, G. F. V.; O'Connor, P. M.; Appella, E. *PNAS* **1997**, *94*(12), 6048–6053.

[5] Fujimoto, H.; Onishi, N.; Kato, N.; Takekawa, M.; Xu, X.; Kosugi, A.; Kondo, T.; Imamura, M.; Oishi, I.; Yoda, A.; others. *Cell Death and Differentiation* **2006**, *13*(7), 1170.

[6] Mrosovsky, N. *Rheostasis. The Physiology of Change;* Oxford University Press: New York, 1990.

[7] Asher, G.; Tsvetkov, P.; Kahana, C.; Shaul, Y. *Genes & Development* **2005**, *19*(3), 316–321.

[8] Asher, G.; Shaul, Y. *Cell Cycle* **2005**, *4*(8), 1015–1018.

[9] Asher, G.; Lotem, J.; Sachs, L.; Shaul, Y. In *Methods in Enzymology*, Vol. 382; Elsevier, 2004; pages 278–293.
