# Supplementary material for: Frequency switching between oscillatory homeostats and the regulation of p53
Source: PLoS One. 2020 May 20;15(5):e0227786. doi: 10.1371/journal.pone.0227786 (PMC7239446; doi:10.1371/journal.pone.0227786)
Supplement: S3 Text — (PDF) [file pone.0227786.s006.pdf]

Supporting Material, File S3 Text

Frequency switching between oscillatory homeostats and the regulation of p53

P. Ruoff[1*], N. Nishiyama[2]
[1]Department of Chemistry, Bioscience, and Environmental Engineering
[2]Division of Mathematical and Physical Sciences
Graduate School of Natural Science and Technology
Kanazawa University, Kanazawa, Japan

*Corresponding author. Address: Department of Chemistry, Bioscience, and Environmental Engineering, University of Stavanger, Stavanger, Norway, Tel.: (47) 5183-1887, E-mail: peter.ruoff@uis.no

Combined m1 and m5 controller motifs and their cooperative and wind-up behaviors

Fig S1 shows the scheme of combined controller motifs m1 and m5 with A being the controlled variable.

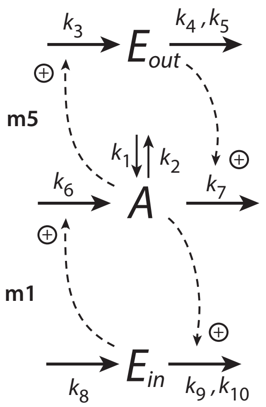

Figure S1. Combined controller motifs m1 and m5.

The rate equations for the combined inflow (m1) and outflow (m5) controllers for nonoscillatory conditions are:

$$\dot{A} = k_1 - k_2 \cdot A + k_6 \cdot E_{in} - k_7 \cdot A \cdot E_{out} \tag{S1}$$

$$\dot{E_{out}} = k_3 \cdot A - \frac{k_4 \cdot E_{out}}{k_5 + E_{out}} \tag{S2}$$

$$\dot{E_{in}} = k_8 - \left(\frac{k_9 \cdot E_{in}}{k_{10} + E_{in}} \right) \tag{S3}$$

The set-points for the two controllers are determined from the steady state condition of the system. From the condition $\dot{E_{out}} = 0$ the set-point of the outflow controller m5 is calculated to be

$$A_{set}^{out} = A_{set}^{m5} = \frac{k_4}{k_3} \tag{S4}$$

while for the inflow controller m3 we have

$$A_{set}^{in} = A_{set}^{m3} = \frac{k_8}{k_9} \tag{S5}$$

At their respective set-points the signs of $\dot{E_{in}}$ and $\dot{E_{out}}$ changes according to m1 and m5 shown in Fig 11.

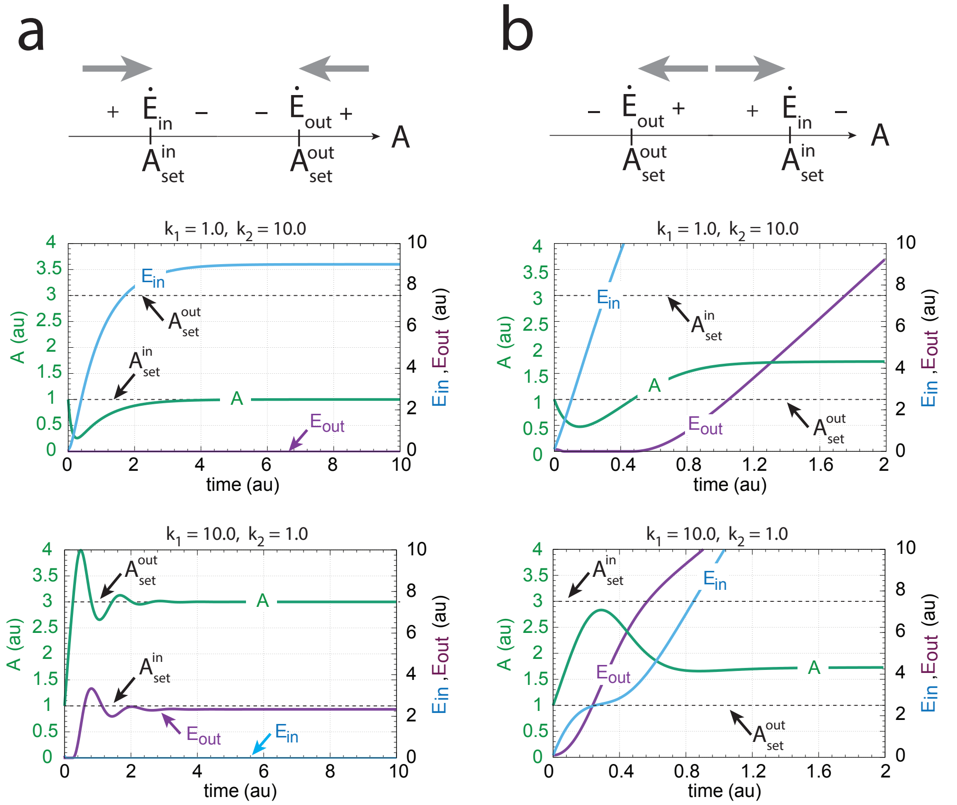

Figure S2. Sign structure of $\dot{E_{in}}$ and $\dot{E_{out}}$ determine whether controllers cooperate or not. Column a: cooperative controllers. Column b: controllers work against each other. Rate constants panels a: k_3=10.0, k_4=30.0, k_5=1 × 10^{-6}, k_6=k_7=1.0, k_8=k_9=10.0, k_{10}=1 × 10^{-6}. Rate constants panels b: k_3=k_4=10.0, k_5=1 × 10^{-6}, k_6=k_7=1.0, k_8=30.0, k_9=10.0, k_{10}=1 × 10^{-6}. k_1 and k_2 are perturbations. Initial concentrations for all calculations: A_0=1.0, $E_{in,0}$=$E_{out,0}$=0.1.

The relative placing of A_{set}^{in} and A_{set}^{out} in the A concentration space determines whether the two controllers cooperate or whether they work against each other.

Fig S2a, upper panel, shows the sign structure when the inflow and outflow controllers cooperate. In this case the negative sign are adjacent to each other and each controller "pulls" A to its set-point dependent whether outflow perturbation k_2 (Fig S2a, middle panel) or inflow perturbation k_1 (Fig S2a, bottom panel) dominates. In this configuration the set-point of the outflow controller, A_{set}^{out}, is larger than the set-point of the inflow controller, $\dot{E}_{in}$.

In case $\dot{E}_{in}$ is larger than A_{set}^{out} the sign structure of $\dot{E}_{in}$ and $\dot{E}_{out}$ leads to controllers which work against each other (Fig S2b, top panel). The controller variables E_{in} and E_{out} show wind-up, i.e., they increase continuously and the steady state of A will lie somewhere between the two controllers' set-points (Fig S2b, middle and bottom panels) dependent on the aggressiveness of the individual controllers. For additional information on wind-up behaviors, see supplementary material of Ref (1).

References

[1] Drengstig, T.; Jolma, I.; Ni, X.; Thorsen, K.; Xu, X.; Ruoff, P. *Biophys J* **2012**, *103*(9), 2000–2010.
