# Supplementary material for: Frequency switching between oscillatory homeostats and the regulation of p53
Source: PLoS One. 2020 May 20;15(5):e0227786. doi: 10.1371/journal.pone.0227786 (PMC7239446; doi:10.1371/journal.pone.0227786)
Supplement: S4 Text — (PDF) [file pone.0227786.s007.pdf]

Supporting Material, File S4 Text

Frequency switching between oscillatory homeostats and the regulation of p53

P. Ruoff[1*], N. Nishiyama[2]
[1]Department of Chemistry, Bioscience, and Environmental Engineering
[2]Division of Mathematical and Physical Sciences
Graduate School of Natural Science and Technology
Kanazawa University, Kanazawa, Japan

*Corresponding author. Address: Department of Chemistry, Bioscience, and Environmental Engineering, University of Stavanger, Stavanger, Norway, Tel.: (47) 5183-1887, E-mail: peter.ruoff@uis.no

Steady state levels of the model as a function of stress level s when feedback loops are non-oscillatory

When the degradation reactions of the controlled variable p53 become first-order with respect to p53, Eq 11 changes to

$$\dot{p53} = k_{31} + \frac{k_1 \cdot s}{K_{as}+s} - \left(\frac{K_{Is}}{K_{Is}+s}\right) + k_{29} \cdot ATM^* + k_6 \cdot Per2 - \left(\frac{K_{I2}}{K_{I2}+Per2}\right) \cdot Mdm2 \tag{S1}$$

and the integral p53-controllers Per2, ATM*, and Mdm2 lose their oscillatory behaviors.

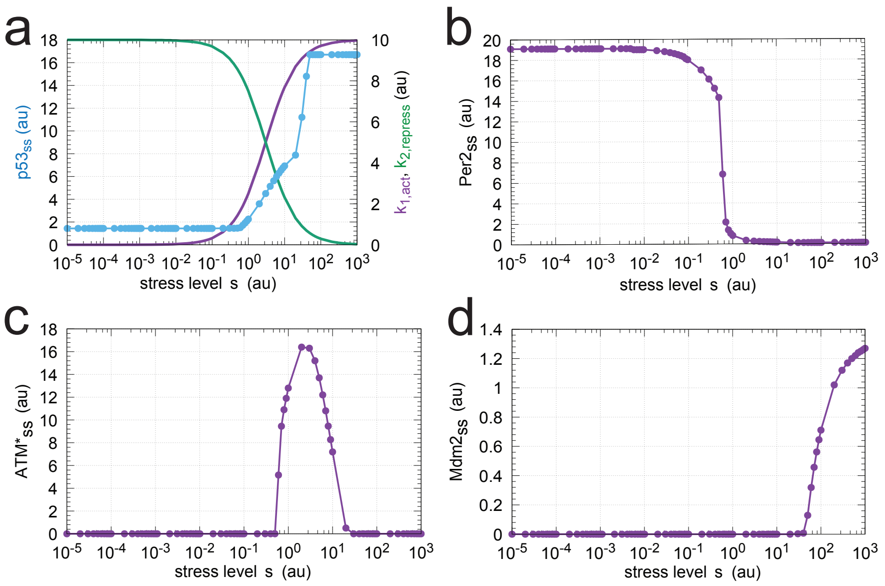

Figure S1. Change of steady state levels of model (Fig 8) as a function of stress parameter s when degradation of p53 is first-order with respect to p53 (Eq S1). Shown levels are (a) p53, $k_{1,act}$, $k_{2,inhib}$; (b) Per2; (c) ATM*; (d) Mdm2. Rate constants as in Fig 9. Initial concentrations: $p53_0$=1.44, $Mdm2_0$=9.44×10^{-8}, $Per2_0$=19.1, $Bmal/Clk_0$=0.7, $Per1_0$=16.27×10^{-2}, $Per1{\cdots}Per2_0$=1.20, $(Per1_2)_0$=7.87×10^{-2}, $(Per2_2)_0$=3.65×10^{-6}, ATM^*_0=2.09×10^{-10}. Step-length (LSODE)=1×10^{-2}, simulation time 3000 time units (h).

Fig S1 shows the steady state levels of p53, Per2, ATM*, and Mdm2 for different stress levels s when using Eq S1 for $\dot{p53}$. All other rate equations remain unchanged. While p53 set-points do not change, controller levels and the rheostatic p53 levels with respect to the control by ATM* are slightly altered in comparison with Fig 9 when controllers are in oscillatory mode. Note that higher stress levels are needed for Mdm2 to take control over p53. Fig S2 shows the steady state levels of p53 and the Per2 as a function of time and the stress level s. Note that Per2 concentrations are non-oscillatory when Per2 takes part in the control of p53. However, when the other feedback loops control p53 Per2 levels are low and become entrained by the circadian Bmal/Clk oscillator.

Figure S2. *(following page)* Non-oscillatory steady state levels of p53 and Per2 at different stress levels s. Parameter values are the same as in Fig S1. (a) s=1×10^{-4}. Per2 determines the concentration of p53. Initial concentrations: $p53_0$=1.44, $Mdm2_0$=9.44×10^{-8}, $Per2_0$=1.91×10^{1}, $Bmal/Clk_0$=0.7, $Per1_0$=6.27×10^{-2}, $(Per1 \cdots Per2)_0$=1.20, $(Per1_2)_0$=7.87×10^{-2}, $(Per2_2)_0$=3.65×10^{-1}, ATM^*_0=2.09×10^{-10}. (b) s=1×10^{-1}. Initial concentrations: $p53_0$=1.44, $Mdm2_0$=9.44×10^{-8}, $Per2_0$=1.80×10^{1}, $Bmal/Clk_0$=0.7, $Per1_0$=6.83×10^{-2}, $(Per1 \cdots Per2)_0$=1.23, $(Per1_2)_0$=9.33×10^{-2}, $(Per2_2)_0$=3.24×10^{-1}, ATM^*_0=2.53×10^{-7}. (c) s=1.0. The ATM* controller has taken over and controls p53 levels. Initial concentrations: $p53_0$=2.25, $Mdm2_0$=1.56×10^{-7}, $Per2_0$=1.29×10^{-5}, $Bmal/Clk_0$=1.34, $Per1_0$=3.98×10^{-1}, $(Per1 \cdots Per2)_0$=5.14×10^{-6}, $(Per1_2)_0$=2.54, $(Per2_2)_0$=1.67×10^{-13}, ATM^*_0=13.37. (d) s=10.0. ATM* still controls p53 levels. Initial concentrations: $p53_0$=6.92, $Mdm2_0$=7.11×10^{-7}, $Per2_0$=1.76×10^{-6}, $Bmal/Clk_0$=1.32, $Per1_0$=2.71×10^{-1}, $(Per1 \cdots Per2)_0$=4.76×10^{-7}, $(Per1_2)_0$=0.875, $(Per2_2)_0$=3.09×10^{-15}, ATM^*_0=7.284. (e) s=100.0. Mdm2 controls the level of p53. Initial concentrations: $p53_0$=16.67, $Mdm2_0$=7.03×10^{-1}, $Per2_0$=6.26×10^{-7}, $Bmal/Clk_0$=1.09, $Per1_0$=1.78×10^{-1}, $(Per1 \cdots Per2)_0$=1.11×10^{-7}, $(Per1_2)_0$=0.286, $(Per2_2)_0$=3.92×10^{-16}, ATM^*_0=1.10×10^{-6}.

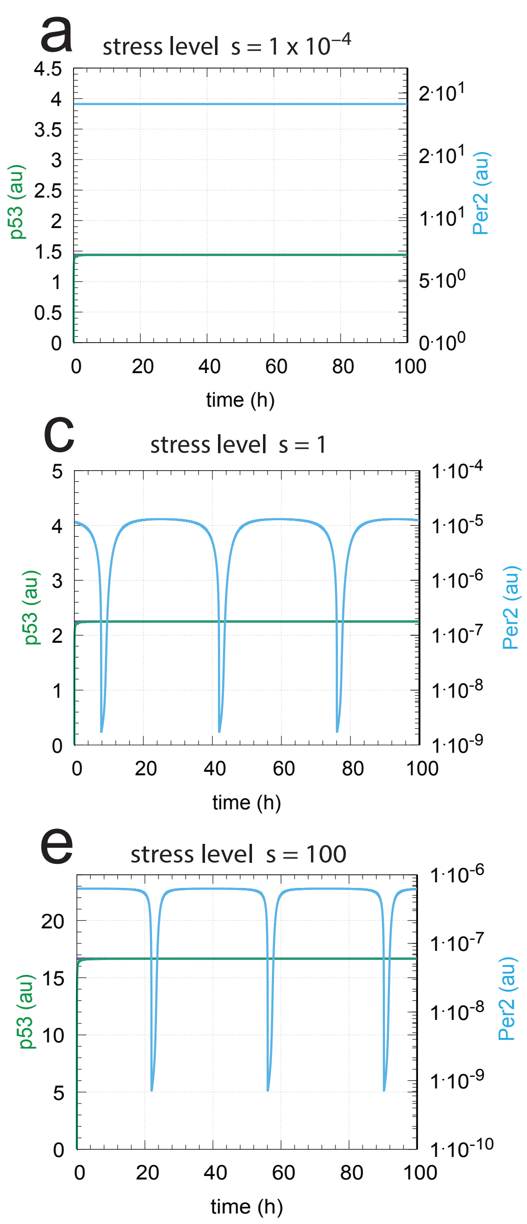

a
stress level s = 1 x 10^−4
b
stress level s = 1 x 10^−1
c
stress level s = 1
d
stress level s = 10
e
stress level s = 100
p53 (au)
Per2 (au)
time (h)
