# Supplementary material for: Frequency switching between oscillatory homeostats and the regulation of p53
Source: PLoS One. 2020 May 20;15(5):e0227786. doi: 10.1371/journal.pone.0227786 (PMC7239446; doi:10.1371/journal.pone.0227786)
Supplement: Attachment — Submitted filename: reply_reviewers.pdf [file pone.0227786.s008.pdf]

Changes made in the revised manuscript

Descriptions of the made changes refer to the submitted marked-up copy of the revised version where changes are outlined in red.

Changes made in the revised manuscript based on the Academic Editor's comments

Academic Editor: "*In particular, the reviewers note several places where the writing could be improved to eliminate ambiguities and inaccuracies. Another example of this issue comes from the abstract: "Mdm2 on its side keeps p53 at a maximum level to avoid premature apoptosis". I encourage the authors to not only respond to the reviewer feedback but also carefully review the entire text before submitting a revised manuscript.*"

Response: Thank you for your comments. We have changed the sentence you refer to, to the following (line 17, outlined in red): *Mdm2 on its side keeps p53 at a high but sub-apoptotic level to avoid premature apoptosis.*

As advised, we have carefully reviewed the entire manuscript and hope the text has become clearer and easier to read.

Changes made in the revised manuscript based on the comments by Reviewer #1

Reviewer #1: "*Define Type 3, Type 1, and Type 5 controllers in the introduction. This terminology could be confusing because, as an example, Uri Alon's Intro to Systems Biology describes totally different motifs with the same 1-8 notation. It's possible that other sources could also define these differently. Further, these motifs only apply to two species systems.*"
Response: We have included the section **The controller motifs used in this study**, where the controller structures 1, 3, and 5 are introduced. In addition, we felt that a section briefly describing how the paper is outlined would be helpful. See the section **Outline of the paper** prior to **Results and discussion**.

Reviewer #1: "Figure 2 is not intuitive and notationally confusing. In the current configuration, it looks like there is interconversion between A and E_in since k_1,k_2 look like a reversible reaction between them. Change this to something like the empty set ø to indicate generation and degradation. Further, you use the Michaelis/Saturation constant K_M multiple times. Denote these as K_(M,i) where i indicates the reaction number. E_out degradation, you list rates k_4 and k_5, but from equation 2, k_5 appears to be a Michaelis constant, similarly with k_10. What does K_I1 denote? Why are letters being used instead of numbers at this point? State that K_M will be omitted in the first model but will be included in later, more complex models.."
Response: We have updated the figure (now Fig 3) such that a possible misunderstanding of A and E_{out} being connected by a reversible reaction is avoided. We feel the empty set is somewhat misleading, because chemical fluxes have different origins and destinations, although they are not specified in the actual figure. Thus, we wish to keep the sources and sinks of chemical fluxes open.

The K_M and $K_M^{'}$ are now mentioned to be used when oscillatory controllers are studied in the following section (see caption of Fig 3).
Concerning the roles of the K_M's (Michaelis constants), K_I's (inhibition constants) and K_a's (activation constant), and why they have been explicitly stated, please see the update of **Materials and methods**, line 63: *However, some K_M's (Michaelis constants), K_I's (inhibition constants) and K_a's (activation constants) are emphasized when they are considered to play a role in the oscillatory and regulatory behavior of controllers.*

Reviewer #1: "The authors rely significantly on self-citations notably 16, 18, and 28. Please acknowledge that these are self-citations (e.g. "In [16], we previously derived…"). This would also clarify why the authors refer to the motifs using such specific terminology which might be unfamiliar to the reader as commented on in major revision 1."
Response: In the section **The controller motifs used in this study** we have now referred to Ref 18, as *"Drengstig et al. [18] previously suggested a set of eight basic 2-species negative feedback structures..."*. As some terminology may appear unfamiliar, we have tried to explain it, as for example in relation to the introduction of the three controllers (Fig 1) in terms of inflow/outflow control. See section **The controller motifs used in this study**.

Reviewer #1: "Graphically, many of the figures appear to be low resolution. Plots are presented as 3D surfaces that are impossible to read rather than pseudo-colormaps. Other figures appear inappropriately vectorized and show distortions upon scaling. Further, the notation and depiction of reaction networks in Figures 2 and 7 is extremely confusing."
Response: Original figures are vector-based pdf files generated by gnuplot and slightly processed by Adobe Illustrator. Figures were then processed by PACE into TIFF format to meet the technical requirements of the journal. For review purposes we have now included the original pdf-figures shown in the marked-up pdf. They look fine in our hands.
Concerning Fig 2 (Fig 3 in revised version) we have explicitly indicated which portion of the combined controllers belongs to motif 5 and which belongs to motif 3. In the manuscript, the figure is directly followed by the rate equations for A, E_{out}, and E_{in}, which should further help to comprehend the network of combined controllers. For Fig 8 (previous Fig 7) we follow the advice of the reviewer (see also below) and use boxes showing which parts constitute motif 1, 3, and 5, and whether they are active or not.

Reviewer #1: "Based on Reference 16, the period derived there is P=2π/(k×k_adapt) (just below Eq. 5 in Ref 16). Unless I am missing something in that source, why does the equation for period involve a square root as if a geometric average was taken? Is there an error in either this manuscript or the author's previous article?"
Response: Thank you for pointing out this discrepancy! This is due to a misprint/error of the harmonic oscillator period in Reference 16. Due to this error, we have added the following sentence (line 158): *Due to a misprint in Ref [16] for the harmonic oscillator solution of controller m5, S1 Text gives the derivations of Eqs 7-9.*

Reviewer #1: "Further, in their derivations the authors assume that K_M, $K_M^{'} \ll$ A, this negates the need to even use a Michaelis-Menten form since the reactions would become pseudo-zero order."
Response: The Reviewer's argument that K_M, $K_M^{'} \ll A$ would negate the need to use a Michaelis-Menten form since the reactions would become pseudo-zero order *neglects the chemical*

constraint that concentrations cannot be negative. The argument is based on a misconception that the chemical behaviors of using (for example) $\dot{A} = -k_2 \cdot A/(K_M + A)$ or $\dot{A} = -k_2$ would be the same when $K_M \ll A$. It is easily seen that this is not the case: In the Michaelis-Menten situation A and $\dot{A}$ will eventually go to zero, while in the $\dot{A} = -k_2$ case A will decrease with constant speed, and continue with negative A-values (which of course needs to be avoided in a chemical situation).

Reviewer #1: "Figure 5 showed fluctuations of A. You were previously able to provide reasonable estimates of the average values of ⟨A⟩, ⟨E_out⟩,and ⟨E_in⟩. Can you analytically estimate or predict the magnitude of fluctuations in this figure?"
Response: A relevant question! Due to the conservative/harmonic nature of the m3 and m5 controllers this is possible. In S1 Text (*Harmonic approximation of period lengths and amplitudes of oscillatory m3 and m5 controllers*) we have given a detailed analysis how period lengths and amplitudes can be analytically determined along with a brief overview in form of two tables. However, in the determination of the A-amplitude for the m3 controller we were not able to get a full analytic description, but a "semi-analytic" one, where the equation

$$c_1 + c_2 \ln(K_{I1} + A) - k_9 A = 0 \tag{1}$$

which determines A_{max} and A_{min} (with A-amplitude being A_{max}-A_{min}) was solved numerically using Matlab.

Reviewer #1: "Amend Figure 6 with an idealized/simplified schematic of Figure 7 showing how all 3 feedback motifs affect P53 similar to Figure 2."
Response: This has now been done. See new Figure 7 with revised caption.

Reviewer #1:"Amend Figure 7 with boxes showing which parts constitute the Motif 1, 3, and 5 feedback systems."
Response: This has now been done. See new Figure 8 with revised caption.

Reviewer #1: "No justification of the rate parameters is provided besides that they exist in a region of parameter space expected to provide oscillatory, integral control. In the methods section, please clearly cite previous models and parameter sets from which this current model was constructed."
Response: Rate constants have been arbitrarily chosen, but the constraints are used that period lengths of the m3 and m5 oscillators should be in the range of experimentally observed values, i.e. ca. 24h for the Per2/m3 oscillator and about 5h for the Mdm2/m5 oscillator. The other constraint used concerns the set-points of all three controllers, which are chosen such that wind-up is avoided. This requires that the set-points are chosen in the following order: $A_{set,m3} < A_{set,m1} < A_{set,m5}$ as indicated in Fig 7.
In chapter **The controller motifs used in this study** we have now added the following paragraph (line 81):
Rate constants have been arbitrarily chosen, but with the constraint that period lengths of the m3 and m5 oscillators are in the range of experimentally observed values, i.e. ca. 24h for the m3 (Per2) oscillator and about 5h for the m5 (Mdm2) oscillator. The other used constraint concerns the chosen set-points for A of the three controllers. Set-points are chosen such that wind-up between controllers is avoided. See the chapter Synergy condition for coupled feedback loops *later in the paper discussing this point.*

Reviewer #1: "As opposed to intrinsic stochasticity in the gene circuit, I think there is the question of parameter heterogeneity. Given that 40% of cells treated with the same radiation dose do not respond in an oscillatory fashion, I feel like a Monte Carlo parameter sweep (even if deterministic integration ignoring intrinsic noise is employed) of certain critical parameters like k_30 should be performed in order to investigate the bifurcation behavior of oscillatory/non-oscillatory cells and to provide potential mechanisms for why this has been experimentally observed."
Response: Since we have shown (Fig 5) that the occurrence of an oscillatory or a nonoscillatory state is closely connected to the degradation kinetics of A (p53), possible differences in the various proteasomal degradation pathways may be an explanation for getting oscillatory or nonoscillatory responses. Mechanistic differences in the proteasomal degradation of p53 may be due to different binding strengths between p53 and the proteasomal proteins/enzymes, thereby resulting in different apparent K_M values that would lead to oscillations in case K_M is low or to a nonoscillatory response when K_M is high.
Due to the reviewer's remarks we have added the following sentences, starting line 418 in the marked-up copy:
The various proteasomal p53 degradation pathways, which have been discovered [37–39], may provide an explanation why some cells show oscillations while others don't. The pathways may differ in their binding strength between p53 and the different proteasomal proteins. In non-oscillatory cells the proteasomal degradation pathway may have a weak binding between p53 and pathway proteins/enzymes (which determine the rate of p53 degradation), and which would lead to a high overall K_M and to approximately first-order p53 degradation kinetics. In oscillatory cells, on the other hand, the dominant proteasomal degradation pathway may have a tighter binding between p53 and proteasomal proteins with the result of overall lower K_M values and close to zero-order degradation kinetics. As long as ATM and Mdm2 contain integral feedback loops one would expect the same regulatory outcome independent of the p53-degradation kinetics, i.e. whether feedback loops are oscillatory or not.*

Reviewer #1: "Figure 11 needs to be significantly reworked for clarity. This is the first time all 8 configurations are introduced in the paper in spite of the fact that the authors expect the reader to be familiar with all of them in the introduction. Further, the sign structure arguments are confusing. It may actually be easier for the author to specify the differential equations and the set point for each configuration so that the reader can algebraically verify the sign structure."
Response: Yes, we agree, and have changed the figure (now Fig 12) to include only the controllers m1, m3, and m5. As suggested by the reviewer we have added the rate equations of the three E_i's, which indicate the sign changes of $\dot{E}_i$ when A is below or above the set-point. The caption has been updated correspondingly.

Reviewer #1: "In Figure 12c, I understand why a signal less than p53-max stress would decrease to p53-min stress since both signs are negative. However, it seems like Per2 would pull the p53 level towards the homeostatic condition while ATM* would pull it to the min damage condition. Why isn't this an example of integral windup?"
Response: Please note that in Fig 13c, when $\dot{Per}2<0$, the controller is inactive. When $\dot{Per}2<0$ the concentration of Per2 will go to very low values (ideally to zero). For concentrations of A larger than p_{53}^{m3} the m3 inflow controller is "happy" (so to speak), i.e. does not add more A to the system. This is the reason why there is no wind-up between m3 and m1.

Minor Revisions:
Reviewer #1: "Approximate ≈ or limit → should be used in Equations 4 and 5 since they are subject to the approximations/limiting arguments described in the preceding paragraph. In Reference 16, which was your own previous paper, you make this distinction."
Response: Yes, we agree. Done.

Reviewer #1: "Figure 3 may be more legible as a 2D pseudo color plot with all 3 side by side. 3D surfaces tend to be harder to read. Also, superimposing two 3D surfaces cannot be read. Figure 5 should be revised as suggested for Figure 3 in minor revision 2. You already appear to have the pseudo-color plots beneath the surfaces. Therefore, you should just use the 2D pseudo-colors."
Response: Fig 3 shows the up- and downregulation of E_{in} and E_{out} together with the corresponding set-points in A. We feel that in the 3-D plots you see the edges/sharpness of the transitions to the set-point plateaus clearly, while in the corresponding 2D-projections the edges of the set-point plateaus are not seen. So, we wish to keep the two figures in their present form.

Reviewer #1: "Grammatical error line 138, change to "degradation of p53, thereby stabilizing p53.""
Response: We have changed the sentence (starting at line 205) to: *In addition, due to its binding to p53, Per2 has been found to inhibit the Mdm2-mediated degradation of p53 which leads to a stabilization of p53.*

Reviewer #1: "Don't denote the radiation damage by k_30. This makes it easily confusable as a reaction rate."
Response: We agree. We have now substituted stress level term k_{30} by "s".

Reviewer #1: "Don't denote Michaelis constants with the same notation as rate constants."
Response: Some K_M's are denoted as K_{M1}, K_{M2}, etc., but please, see our description in "Materials and methods" starting at line 61.

Reviewer #1: Grammatical error line 177. Change to "In stressed cells, several..."
Response: Done. See line 245.

Reviewer #1: "The graphical resolution of Figure 8 looks low quality. It seems like things were scaled up inappropriately. Throughout Figure 8, the resolution of the graphs near the phase transition is insufficient. While the Circadian and high stress states are oversampled, the region around the two phase transitions could benefit from enhanced simulation resolution."
Response: As commented before, in our hands original figures (vector-based pdfs) and submitted TIFF-files look fine and are sharp. Perhaps this is a journal issue? We have mentioned it in our cover letter to the editor.
Concerning the resolution of Fig 8 (now Fig 9) we have increased the resolution across the transitions showing that some of the controller properties show practically discontinuous transitions. The inset in previous Fig 8c has been removed, as there is no more need for it.

Changes made in the revised manuscript based on the comments by Reviewer 2

Reviewer #2: "1-The authors should rework in depth the structure and the writing of the manuscript. The biological data used to build the model, the assumptions made, and the analysis of the model outcome should be more clearly delineated. Moreover, some parts of the paper resembles a catalogue of descriptions (for example p.9) which make the manuscript unclear and hard to follow. The description of the model and its analysis are not separated clearly enough, which makes the outcome of the work confusing."
Response: We have now revised the manuscript significantly which includes a more detailed description of the three controller motifs (see chapter *The controller motifs used in this study* under *Materials and methods*) together with a chapter *Outline of the paper* to give an overview how the manuscript is structured. Fig 7 and Fig 8 have undergone major revisions to make the contributions of the different controllers under different stress levels more clear.

Reviewer #2: "2- Some statements need more explanations/justifications. For example, there is a lack of explanations of the type of functions (Michaelis-Menten, linear, product of Michaelis-Menten,. . .) used to model the processes appearing in the differential equations. Moreover, some biological data used to built the model is lacking (role of Bmal/Clk, dimerization of Per1 and Per2)."
Response: We have included a more detailed description of the conditions employed on the controllers. In line 124-133 we describe the two distinct conditions for controller accuracy and for generating oscillations while still imposing controller function (i.e.,keeping homeostasis). See section outlined in red starting with (line 124)
There are two separate and independent conditions which have been applied on the controllers...
We have also added a paragraph on the circadian components added in the model. See paragraph outlined in red starting with (line 283):
Per2, which takes part in the regulation of p53 in unstressed cells, is an important component of the mammalian circadian clock...
In this paragraph we relate the circadian part of the model to biological data/findings.

Reviewer #2: "3- The level of English should be strongly improved. For example: "(K_m not used)" l.81; "we arrive at" l.171 ; "the other rate equations are" l.205; "has apparently the function" l.291,..."
Response: We have gone thoroughly through the manuscript to improve the presentation and have made some adjustments including the cases mentioned by the reviewer (see sentences starting lines 129, 273, and 374). However, we don't see anything wrong in the sentence starting line 239: *Based on these observations, we arrive at....* We use here *arrive at* with the following meaning (citing the dictionary): "(arrive at) reach (a conclusion or decision): they arrived at the same conclusion."
